# Supplementary material for: Titles and abstracts of scientific reports ignore variation among species
Source: eLife. 2014 Dec 24;3:e05075. doi: 10.7554/eLife.05075 (PMC4275570; doi:10.7554/eLife.05075)
Supplement: Table 1—source data 1. — DOI: http://dx.doi.org/10.7554/eLife.05075.003 [file elife05075s001.docx]

**Table 1: Source material for eLife papers (published between July 1, 2014 and September 16, 2014)**

| Date | # | Title | Is the species name mentioned in the | | | | Species studied |
| --- | --- | --- | --- | --- | --- | --- | --- |
|  |  |  | Title | Impact  statement | Abstract | Digest |  |
| July 1 | 1 | Interactions with RNA | No | No | No | No | Hela cells |
|  | 2 | Transport of soluble | No | No | No | No | Hela & other human cells |
|  | 3 | Structure of cellular ESC | No | No | No | No | Hela & other human cells |
|  | 4 | Cell autonomous regul | No | No | Yes | Yes | Mice |
|  | 5 | Natural variation reveals | No | Yes | Yes | Yes | Arabidopsis |
|  | 6 | Loss of multifunctional R | No | No | No | No | Mice |
|  | 7 | Sensory experience | No | Yes | Yes | Yes | Mice |
|  | 8 | Coordinated control of | No | No | No | No | Human foreskin fibroblsts |
|  | 9 | Cyclin D activates | No | No | No | No | Human & mouse cell lines |
|  | 10 | Cell elongation is regulate | Yes | Yes | Yes | Yes | Arabidopsis |
|  | 11 | Samonella & enteric fever | Yes | No | Yes | Yes | Samonella |
| July 8 | 1 | Core promoter factor | No | No | Yes | Yes | Mice |
|  | 2 | The pseudo GTPase | Yes | No | No | No | Human kinetochore |
|  | 3 | Induction of homologous | No | No | No | Yes | Chicken & Mouse |
|  | 4 | High resolution mapping | Yes | Yes | Yes | Yes | Swordtail fish |
|  | 5 | Requirement of Smurf- | No | No | No | No | Mice |
|  | 6 | Cryo-EM structure | Yes | Yes | Yes | Yes | Plasmodium malariae |
| July 15 | 1 | Designed sheet peptid | No | No | No | No | Hela cells |
|  | 2 | The quantitative architect | No | Yes | Yes | Yes | Hela cell centromeres |
|  | 3 | The transcription factor | No | No | Yes | Yes | Mouse ES cells |
|  | 4 | Single molecule tracking | No | No | No | No | Human osteosarcoma cells |
|  | 5 | Thrombospondin-4 cont | No | No | Yes | Yes | Zebrafish |
|  | 6 | Hidden synaptic differenc | No | No | Yes | Yes | Mollusc ( Sea slug) |
|  | 7 | PDF neuron firing | Yes | Yes | Yes | Yes | Drosophila |
|  | 8 | G protein-coupled | No | No | No | No | Human cancer cells |
|  | 9 | Allosteric inhibition | No | No | No | No | Rat & Human cancer cells |
|  | 10 | Auxin efflux | No | Yes | Yes | Yes | Arabidopsis & Xenopus |
|  | 11 | Three pools of plasma | No | No | No | No | Human SV-589 cells & Hamster Cho cells |
| July 22 | 1 | Host-induced bacterial | Yes | Yes | Yes | Yes | Arabidopsis |
|  | 2 | Role of photorespiration | Yes | No | No | Yes | Flaveria (Sunflower) |
|  | 3 | Rett-causing mutations | Yes | Yes | Yes | Yes | Transgenic mice |
|  | 4 | Molecular mechanism | NOT RELEVANT |  |  |  |  |
|  | 4 | Global distribution | NOT BIOLOGICAL |  |  |  |  |
|  | 4 | C. elegans male sensory | Yes | Yes | Yes | Yes | C. Elegans |
|  | 5 | Molecular model | NOT BIOLOGICAL |  |  |  |  |
|  | 5 | Optimal multisensory | No | Yes | No | Yes | Normal or corrected humans |
| July 29 | 1 | Integrated action of | Yes | Yes | Yes | Yes | Mice |
|  | 2 | GSK-3 signalling in devel | No | No | Yes | Yes | Mice |
|  | 3 | Flagellar synchronization | No | No | Yes | Yes | Volvox green algae |
|  | 4 | Enhancement of encoding | No | Yes | Yes | Yes | Mice |
|  | 5 | Intact protein folding | No | No | No | No | Hela & Hek cells |
|  | 6 | A dynamin-1 dynamin3 | No | No | No | No | mice |
|  | 7 | A role for descending aud | Yes | Yes | Yes | Yes | Songbird Zebra finch |
|  | 8 | An atomic resolution | NOT RELEVANT |  |  |  |  |
|  | 8 | Contribution of correlat | No | No | No | No | Monkeys |
|  | 9 | A comprehensive search | No | No | No | No | HEK 293 cells, Xenopus oocytes |
|  | 10 | Specific Polar Subpopulat | No | No | Yes | Yes | Mouse brain |
|  | 11 | A single vertebrate DNA | No | Yes | Yes | Yes | Moths & butterflies |
|  | 12 | Inherent mutational | No | No | No | No | Human cells |
| August 5 | 1 | Protein kinase C is a calci | No | No | Yes | No | Mice |
|  | 2 | Nucleus reuniens of the | No | No | Yes | Yes | Rat |
|  | 3 | Concerning RNA guided | NOT RELEVANT |  |  |  |  |
|  | 3 | Resting state functional | No Yes | No | Yes | No | Normal human subjects |
|  | 4 | Natural epigenetic poly | Yes | Yes | Yes | Yes | Arabidopsis |
|  | 5 | Hippo effector Yorkie | No | No | Yes | Yes | Drosophila |
|  | 6 | Loss of Cdc42 leads to | No | Yes | No | Yes | Mouse |
|  | 7 | Disparate substrates for | Yes | Yes | Yes | Yes | Monkey |
|  | 8 | Prediction & characteriz | NOT RELEVANT |  |  |  |  |
|  | 8 | Quantitative determinant | Computational Model |  |  |  |  |
| Aug 12 | 1 | Laser ablation of Dbx1 | Yes | No | Yes | Yes | Neonatal mice |
|  | 2 | Human promoter area | Yes | No | Yes | Yes | Human volunteers |
|  | 3 | Quantitative proteomic | Yes | No | Yes | Yes | Yeast |
|  | 4 | Serum Amyloid A is a | No | No | Yes | Yes | Mice mainly and human |
|  | 5 | Transcription factor NRS | No | No | No | Yes | Rats |
|  | 6 | Large-scale filament | NOT RELEVANT |  |  |  |  |
|  | 6 | LARP7 suppresses P-TEF | No | No | No | No | Human and mouse breast cancer cells |
|  | 7 | Direct measurement | NOT RELEVANT |  |  |  |  |
|  | 7 | High temperature sensiti | NOT RELEVANT |  |  |  |  |
|  | 7 | Insect endosymbiont | Yes | Yes | Yes | Yes | Spiroplasma |
|  | 8 | A structural model | NOT RELEVANT |  |  |  |  |
| Aug 19 | 1 | Shared mushroom body | Yes | Yes |  |  | Drosophila |
|  | 2 | A suppression hierarchy | Yes | Yes | Yes | Yes | Drosophila |
|  | 3 | Quantitative analysis of | No | No | No | Yes | Mouse GIRK2 in yeast cells |
|  | 4 | Master cell cycle regulato | No | No | No | No | Mouse retinal cells |
|  | 5 | The metal transporter | Yes | Yes | Yes | Yes | Drosophila |
|  | 6 | Orbital frontal neurons | No | No | Yes | Yes | Rats |
|  | 7 | Redox signaling via | No | No | No | Yes | Yeast cells |
|  | 8 | Autism & attention deficit | Yes | Yes | Yes | Yes | Children of alcoholics |
|  | 9 | Adult born granule cells | No | No | No | No | Young adult rats |
|  |  |  |  |  |  |  |  |
| Aug 26 | 1 | Adipocyte ALK7 links | No | No | Yes | Yes | Adult mice |
|  | 2 | Methylation of histone | No | No | Yes | Yes | Tetrahymena |
|  | 3 | Mismatch repair deficienc | No | No | No | No? | Human tumor & normal tissue |
|  | 4 | Calmodulin-controlled | No | No | No | No | Mouse beta cell tumor |
|  | 5 | H3K27 modifications | Yes | No | Yes | Yes | Drosophila |
|  | 6 | Structural basis of nucleo | NOT RELEVANT |  |  |  |  |
|  | 6 | Bidirectional helical motil | No | No | No | No | Yeast |
|  | 7 | Lhx1 maintains synchron | No | No | Yes | Yes | Mice |
| Sept 2 | 1 | DNA binding polarity | NOT RELEVANT |  |  |  |  |
|  | 1 | Buffered qualitative | NOT RELEVANT |  |  |  |  |
|  | 1 | Prion propogation can | No | Yes | Yes | Yes | E. coli |
|  | 2 | Mechanistic insight into | NOT RELEVANT |  |  |  |  |
|  | 2 | Microtubules provide | No | No | No | Yes | Epithelial cells of fly wings |
|  | 3 | Nuclear envelope protein | No | No | No | No | Flies & Mice |
|  | 4 | Assembly principles of a | Yes | Yes | Yes | Yes | E coli |
|  | 5 | A tethered delivery mech | NOT RELEVANT |  |  |  |  |
|  | 5 | A long non-coding RNA | Yes | Yes | Yes | Yes | Hela Cells not mentioned |
|  | 6 | RNA-guided assembly of | NOT RELEVANT |  |  |  |  |
|  | 6 | The Drosophila F-box pro | Yes | No | Yes | Yes | Drosophila |
| Sept 9 | 1 | Histone supply regulates | No | No | Yes | Yes | Drosophila |
|  | 2 | MicroRNA -mediated | No | No | No | No | Human cancer cell lines |
|  | 3 | Molecular assembly of th | NOT RELEVANT |  |  |  |  |
|  | 3 | Coding of stimulus streng | Yes | No | Yes | Yes | Mice |
| Sept 16 | 1 | Extensive translation of | NOT RELEVANT |  |  |  |  |
|  | 1 | Long non-coding RNAs | NOT RELEVANT |  |  |  |  |
|  | 1 | A receptor-like kinase | No | Yes | Yes | Yes | Plant roots (Arabidopsis) |
|  | 2 | Translation elongation | No | No | No | Yes | Hela & mouse MEFs |
|  | 3 | Ecology & evolution of | Yes | Yes | Yes | Yes | SUP05 bacteria & viruses |
|  | 4 | Ribosomal protein S27- | No | No | Yes | Yes | Mice |
|  | 5 | Selection of chromosomal | No | No | Yes | Yes | Yeast |
|  | 6 | Reconceiving the hippo | No | No | Yes | Yes | Rats |
|  | 7 | Epsin deficiency impairs | No | No | Yes | Yes | Mice fibroblasts of |
|  | 8 | A RanGTP-independent | No | No | Yes | Yes | Yeast |
